# Structural efficiency of blast furnace steel slag aggregate concrete for strengthening RC flat slabs

**DOI:** 10.1038/s41598-026-69345-1

**Published:** 2026-09-10

**Authors:** Mohamed Ahmed, Mohamed A. Abouelnour, Mohamed Abou-Elmaaty Amin, Mahmoud Zaghlal, Eman Elshamy

**Affiliations:** 1 Structural Engineering Department, Zagazig University, Zagazig, 44519 Egypt; 2 Civil Engineering Department, Faculty of Engineering, Fayoum University, Fayoum, Egypt

**Keywords:** Blast furnace slag, RC flat slab strengthening, ANSYS finite element analysis, Sustainable concrete, Engineering, Materials science

## Abstract

This study investigates blast furnace steel slag concrete as a sustainable strengthening layer for reinforced concrete (RC) flat slabs, with natural aggregates fully replaced by slag in the overlay. Five slabs (1500 × 1500 × 100 mm) were tested under static loading and compared against FRP, steel fiber-reinforced concrete, and high-performance concrete strengthening methods. The slag overlay increased ultimate load by 29.9% and first-crack load by 15.7%, while reducing ultimate deflection by 56.4% compared to the control slab. A validated ANSYS finite element model was used to conduct 22 parametric configurations examining overlay thickness, slab aspect ratio, and bottom reinforcement corrosion. Results show that increasing the aspect ratio from 1.2 to 2.0 reduces ultimate load by 73%, and 25–100% bottom mesh corrosion reduces ultimate load from 233 kN to 61.5 kN. An overlay of 20–30% of slab thickness is recommended as the optimal strengthening configuration.

## Introduction

Reinforced concrete (RC) flat slabs are extensively employed in residential, commercial, and industrial structures due to their architectural flexibility, efficient load distribution, and ease of construction. Despite their widespread use, RC flat slabs are susceptible to serviceability and structural deficiencies, primarily due to the development of cracks in the tensile zone. Tensile cracks may initiate as a result of insufficient reinforcement, excessive service loads, environmental degradation, poor workmanship, and long-term effects such as shrinkage and creep. Once these cracks appear, they can reduce slab stiffness, increase deflection, and in severe cases, trigger punching shear failures or partial collapse. Previous studies have emphasized that tensile-zone cracking is one of the principal causes of premature deterioration in RC slabs and a key factor motivating the need for strengthening interventions^1–4^. Cracking in the tensile zone not only reduces the effective structural section but also allows crack widths to widen progressively, causing stiffness degradation. In long-span flat slabs or slabs subjected to heavy imposed loads, the tensile reinforcement alone may not suffice to carry additional stress. Furthermore, the propagation of flexural cracks accelerates the ingress of moisture, chlorides, and other aggressive agents, promoting corrosion of reinforcement and further weakening the slab system. Recent investigations have shown that tensile-zone cracking significantly affects both serviceability and punching resistance, which is critical for flat slab systems^2,3,5^. Hussein et al. (2023) reported that early tensile cracking restricts rotational capacity and accelerates the transition toward brittle punching failures^5^. Similarly, Karim et al. (2020) demonstrated that severe flexural cracking can reduce effective stiffness by more than 40%, compromising both service and ultimate limit states^6^.The growing interest in modern strengthening techniques aims to restore structural performance and prolong service life.

Over the past decade, a variety of strengthening methods have been proposed to improve tensile behavior in RC slabs, each with specific advantages and limitations^7–9^. These include increasing cement content in the strengthening overlay^10^, externally bonded fiber-reinforced polymer (FRP) sheets^11,12^, the addition of steel fibers to the strengthening concrete^13^, and the use of sustainable industrial by-products^14,15^ such as ground granulated blast furnace slag (GGBS)^16^. One of the traditional approaches to strengthen RC slabs involves applying a cement-rich or high-performance concrete overlay on the tension face. This method increases thickness and improves flexural stiffness and load-carrying capacity. Recent studies demonstrate that overlay systems^17^, particularly when high-performance^18^ or silica-modified concrete^19^ is used, can significantly enhance slab performance. Sharaf Aldeen and Abdellatif (2024) indicated that well-bonded overlays improved flexural capacity by up to 30% due to better tensile-zone confinement^20^. Al-Zuhairi et al. (2020) reported that increasing cement content in overlays substantially improved crack resistance and delayed the formation of plastic hinges^21^. Mohan et al. (2025) highlighted the importance of surface preparation to prevent debonding due to shrinkage stresses^22^. Sharaf Aldeen and Abdellatif (2024) further confirmed through nonlinear 3D finite element modeling that concrete overlays can modify crack propagation patterns and restore stiffness effectively^20^.

Externally bonded FRP systems are widely adopted for flexural strengthening due to their high tensile strength, corrosion resistance, and ease of installation^23^. FRP materials, including carbon (CFRP)^24^, glass (GFRP)^25^, and basalt (BFRP)^8^, have been extensively investigated, demonstrating significant improvements in ultimate load and crack control^26^. Makhlouf et al. (2024) reported flexural capacity gains up to 67% when using novel anchoring techniques for FRP strengthening^27^. Osman (2023) compared different FRP strengthening guidelines and highlighted the sensitivity of FRP systems to anchorage failure and surface preparation^28^. Al Kallas et al. (2023) observed that FRP delamination can occur under cyclic or impact loading if installation is improper^29^. Despite these limitations, FRP remains attractive due to its high strength-to-weight ratio and relatively rapid installation. Integrating steel fibers in the strengthening layer is a promising method for improving tensile behavior. Steel fibers enhance ductility, improve crack control, and increase post-cracking tensile strength. Zhao et al. (2023) observed that steel fibers delay crack formation and improve energy absorption under flexural loading^6^. Rasheed and Mohammed (2024) demonstrated improved residual strength in slabs with combined GFRP and steel reinforcement under fire exposure^30^. El Hamahmy (2025) emphasized that steel fibers provide superior toughness and cracking resistance, making them ideal for high-ductility applications^2^. Challenges include reduced workability and the need for precise quality control during mixing.

The construction industry is increasingly under pressure to adopt sustainable practices by reducing reliance on virgin natural resources and minimizing industrial waste. The recycling of industrial by-products and waste materials as concrete constituents has demonstrated significant potential in improving mechanical performance, durability, and radiation shielding properties while simultaneously reducing environmental impact^31,32^. Replacing natural aggregates or cement with such by-products not only diverts waste from landfills but also enhances concrete microstructure through supplementary chemical reactions, contributing to circular economy principles in construction^33,34^. In line with sustainable construction goals, industrial by-products such as GGBS are increasingly used as partial or full cement replacements in strengthening layers. GGBS improves durability, reduces environmental impact, and enhances long-term mechanical performance through latent hydraulic reactions. Yang et al. (2020) evaluated the strength development in slag-based concrete and confirmed significant enhancement of tensile behavior^35^. Amin (2020) investigated RC beams strengthened with blast furnace slag and reported improved flexural performance and crack control^36^. Osman and Irshidat (2025) demonstrated that GGBS-based geopolymer mortars can effectively strengthen pre-loaded RC beams, recovering flexural capacity^1^. Hassan and Altai (2024) highlighted the benefits of GGBS in high-strength RC beams, improving both load capacity and service life^37^. The incorporation of alternative waste-derived materials into concrete mixes has also gained considerable traction as part of broader sustainability efforts. For instance, the combined use of waste glass cullet and snail shell powder has been shown to influence both workability and compressive strength characteristics of concrete, demonstrating that unconventional by-products can be valorized within cementitious systems^38^. Similarly, the use of metakaolin as a supplementary cementitious material in laterite rock aggregate concrete has demonstrated meaningful strength optimization potential, reinforcing the case for calcined clay-based SCMs as sustainable cement replacements in structural applications^39^. These findings collectively underscore that the field of sustainable concrete is rapidly expanding beyond conventional SCMs such as GGBS and fly ash, encompassing a wider palette of industrial and agricultural waste streams who’s mechanical and durability contributions are increasingly well-documented.

Beyond its use as a supplementary cementitious material, steel slag has also been extensively investigated as a direct replacement for natural aggregates in concrete. Mitwally et al.^40^ examined steel slag as a partial replacement for coarse aggregate, reporting that a 30% replacement level achieved optimal compressive and flexural strength alongside favorable workability, and proposed predictive relationships between compressive, flexural, and splitting tensile strengths to guide concrete mix design. Cheng et al.^41^ investigated the sulfate erosion resistance of steel slag coarse aggregate concrete, finding that a 60% substitution rate optimally enhanced compressive strength (up to 20.7%) and durability, attributing this improvement to active components in steel slag promoting hydration products that strengthen aggregate–paste bonding, while excessive substitution (90%) caused loosening and reduced durability. Ho et al.^42^ studied steel slag coarse aggregate concrete (0–100% replacement) under elevated temperatures up to 1000 °C, showing that higher slag replacement improved heat-insulation capacity through increased wet packing density and lower thermal conductivity, with full replacement yielding residual compressive strength comparable to conventional concrete at temperatures ≥ 600 °C. Keertan et al.^43^ evaluated high-strength fiber-reinforced concrete (M70 grade) with coarse aggregate replaced by steel slag (40–50%) combined with 1% steel fibers, finding that 50% replacement produced optimal compressive and split tensile strength gains, attributed to a denser, less porous C-S-H microstructure. Collectively, these studies confirm that steel slag can serve as a viable coarse aggregate replacement, offering comparable or enhanced mechanical performance, improved durability under aggressive exposure, and favorable thermal behavior. However, existing research has predominantly focused on partial replacement levels and material-scale mechanical characterization, with limited investigation into full (100%) aggregate replacement at the structural component scale — a gap this study addresses by evaluating blast furnace steel slag as a complete coarse and fine aggregate replacement in RC flat slab strengthening applications.

This study presents a comprehensive experimental and numerical investigation of RC flat slabs strengthened with blast furnace steel slag concrete, examining critical performance parameters including crack propagation patterns, load-deflection response, structural stiffness, energy dissipation capacity, and failure mechanisms. The experimental program comprises five full-scale slab specimens: one control specimen and four slabs strengthened using alternative techniques, FRP sheets, steel fiber-reinforced concrete, high-performance concrete, and blast furnace steel slag concrete overlay. Complementary numerical modeling is performed using ANSYS finite element software to simulate 22 parametric slab configurations, systematically investigating the influence of strengthening layer thickness, slab aspect ratio (rectangularity), and varying degrees of bottom reinforcement corrosion. The primary objective is to establish design-oriented guidelines for sustainable and effective strengthening solutions in the tensile zones of RC slabs. Specifically, this research aims to: (1) behavior of RC flat slabs retrofitted with blast furnace steel slag concrete; (2) conduct comparative performance assessments between slag-strengthened slabs and conventional strengthening methodologies, including FRP composite systems, high-strength concrete overlays, and steel fiber-reinforced concrete; (3) validate experimental findings through numerical simulation and establish model reliability; (4) quantify the effects of geometric parameters, strengthening layer dimensions, and reinforcement deterioration on flexural performance characteristics; and (5) develop practical, implementation-focused design recommendations for slag-based strengthening systems in RC slab applications. Through these research objectives, this investigation contributes to the advancement of sustainable construction practices while simultaneously enhancing the serviceability, load-carrying capacity, and long-term durability of existing RC slab structures. The novelty of this study lies in three aspects that distinguish it from prior GGBS research: (1) Unlike studies employing GGBS as cement replacement^1,35–37^, this study uses blast furnace steel slag as a complete replacement for both coarse and fine natural aggregates in the strengthening overlay, fundamentally changing the mechanical and sustainability profile of the system. (2) This is the first study to systematically compare slag-aggregate concrete overlays with FRP, steel fiber-reinforced concrete, and high-performance concrete as strengthening methods for RC flat slabs under a standardized test protocol. (3) The parametric numerical investigation addresses variables — slab aspect ratio, overlay thickness ratio, and bottom mesh corrosion — that have not previously been studied in combination with slag-based strengthening systems.

## Experimental works

### Materials properties

#### Materials composing the control mix

The materials for the baseline reinforced concrete slabs (control specimens prior to strengthening layer application) comprised Portland cement (CEM I 42.5 N) conforming to ASTM C150^44^ to ensure consistent quality and strength development, natural fine aggregate (NFA) with maximum particle size of 4.75 mm and specific gravity of 2.5, natural coarse aggregate (NCA) ranging from 4.75 mm to 22.4 mm with specific gravity of 2.65, and potable tap water for achieving requisite workability. Selected specimens incorporated coarse and fine steel slag aggregates as complete replacements for natural aggregates to evaluate sustainable concrete alternatives. High-strength steel reinforcement bars (grade 28/45, Ø 8 mm diameter) were employed in dual-layer mesh configurations for both top and bottom reinforcement. The control concrete mixture was proportioned in accordance with American Concrete Institute guidelines (ACI 2011^45^, with mix compositions calibrated to achieve target workability, compressive strength, and durability parameters as detailed in Table 1, thereby ensuring experimental uniformity and reproducibility across all test specimens.

**Table 1:** NSC mix design proportion (kg/m^3^).

Normal concrete				Strengthening layer (Slag concrete)			
Cement	NCA	NFA	Water	Cement	NCA	NFA	Water
350	1235	620	180	350	1520	760	180

#### Steel reinforcement

All experimental slabs were reinforced using two layers of steel bars, with Ø 8 mm bars employed for both transverse and longitudinal reinforcement. The steel reinforcement was selected to ensure adequate flexural and shear performance of the slabs under applied loading. The mechanical properties of the steel bars, including yield strength, ultimate strength, and elongation, were determined in accordance with ASTM A370^46^, as summarized in Table 2. The steel area provided per direction and layer was calculated based on the reinforcement configuration of 5Ø8 mm bars per metre run, using the cross-sectional area of a single Ø8 mm bar (As, bar = π × 8²/4 = 50.24 ≈ 50.2 mm²), giving a total steel area of As = 5 × 50.2 ≈ 251 mm²/m. Based on this reinforcement area of 251 mm²/m and an effective depth of 80 mm (control slab, 100 mm thickness with 20 mm cover), the resulting reinforcement ratio is ρ = As/(b×d) = 251/(1000 × 80) ≈ 0.31%.

**Table 2:** Steel reinforcement properties.

bar No.	Actual diameter (mm)	Actual area (mm^2^)	fy (*N*/mm^2^)	fu (*N*/mm^2^)	% elongation
8	8	50.2	280	492	19.50

The reported area of 50.2 mm² corresponds to the cross-sectional area of a single Ø8 mm reinforcement bar (π × 8²/4 = 50.24 ≈ 50.2 mm²).

#### Cement

The cement used in all concrete mixes was Ordinary Portland Cement (CEM I 42.5 N). To ensure compliance with quality standards, a series of tests were conducted on the cement in accordance with ASTM C150^44^. These tests included assessments of setting time, fineness, specific gravity, and soundness, which are critical for achieving the desired strength and durability of the concrete. The measured physical properties of the cement, as obtained from the laboratory tests, are summarized in Table 3.

**Table 3:** Physical, mechanical and chemical properties of used cement.

Property		Result
Specific gravity		3.17
Fineness		2000 cm^2^/gm
Initial setting time		85 min
Final setting time		180 min
Compressive strength	3 days	205 Kg/cm^2^
	7 days	300 Kg/cm^2^
	28 days	435 Kg/cm^2^
Soundness		1 mm
SiO_2_		20.57
CaO		63.48
Al_2_O_3_		3.27
Fe_2_O_3_		4.93
MgO		2.15
MnO		0.11
SO_3_		1.98
Na_2_O		0.39
K_2_O		0.62
P_2_O_5_		0.05
Cl		0.01
TiO_2_		0.25
LOI		2.08

#### Blast furnace steel slag

The slag used in this study is air-cooled blast furnace slag, sourced as raw lump material from Suez Steel Co. (Solb Misr), one of Egypt’s leading steel producers by production capacity. The raw lumps were subsequently crushed and screened into coarse (> 4.75 mm) and fine (< 4.75 mm) fractions for use as a 100% replacement of the natural coarse and fine aggregates, respectively — an identification consistent with the chemical composition reported in Table 4. The raw slag exhibited significant density variations across the sampled batch, ranging from lightweight, porous particles to dense particles with minimal voids. Based on these observations, the slag aggregates sourced from the steel plant were found to comprise two distinct types, depending on the cooling method applied during production. The first type is heavyweight slag (SH), produced through air-cooling, in which the molten slag is cooled slowly by exposure to air; this gradual cooling allows the material to crystallize into a dense, structurally solid particle with minimal internal voids, resulting in a higher density. The second type is lightweight slag (SL), produced through rapid water-quenching, in which the molten slag is cooled almost instantaneously; this rapid cooling prevents crystallization and traps air/steam voids within the particle, yielding a porous, glassy structure and correspondingly lower density compared to the air-cooled type. In addition, the steel plant also produced a mixed slag (SM) fraction, consisting of a blend of both air-cooled and water-quenched particles.

**Table 4:** Chemical composition of slag.

Component	CaO	SiO_2_	Al_2_O_3_	MgO	FeO	S	MnO	*P*_2_O_5_
(%)	41.7	33.8	13.4	7.4	0.4	0.8	0.3	> 0.1

For the purposes of this study, the heavyweight, air-cooled slag (SH) fraction was selected and used as the aggregate replacement, as its density and physical characteristics (Table 5) were found to be more comparable to those of natural aggregates, making it more suitable for achieving concrete properties consistent with conventional natural-aggregate concrete.

**Table 5:** Physical and durability properties of the slag aggregate.

Bulk density (kg/m^3^)	Specific gravity	Absorption (%)	Abrasion resistance (%)	Soundness (%)	Free CaO (%)	Free MgO (%)
1450	2.68	1	25	0.3	2	0.5

**Fig. 1:**
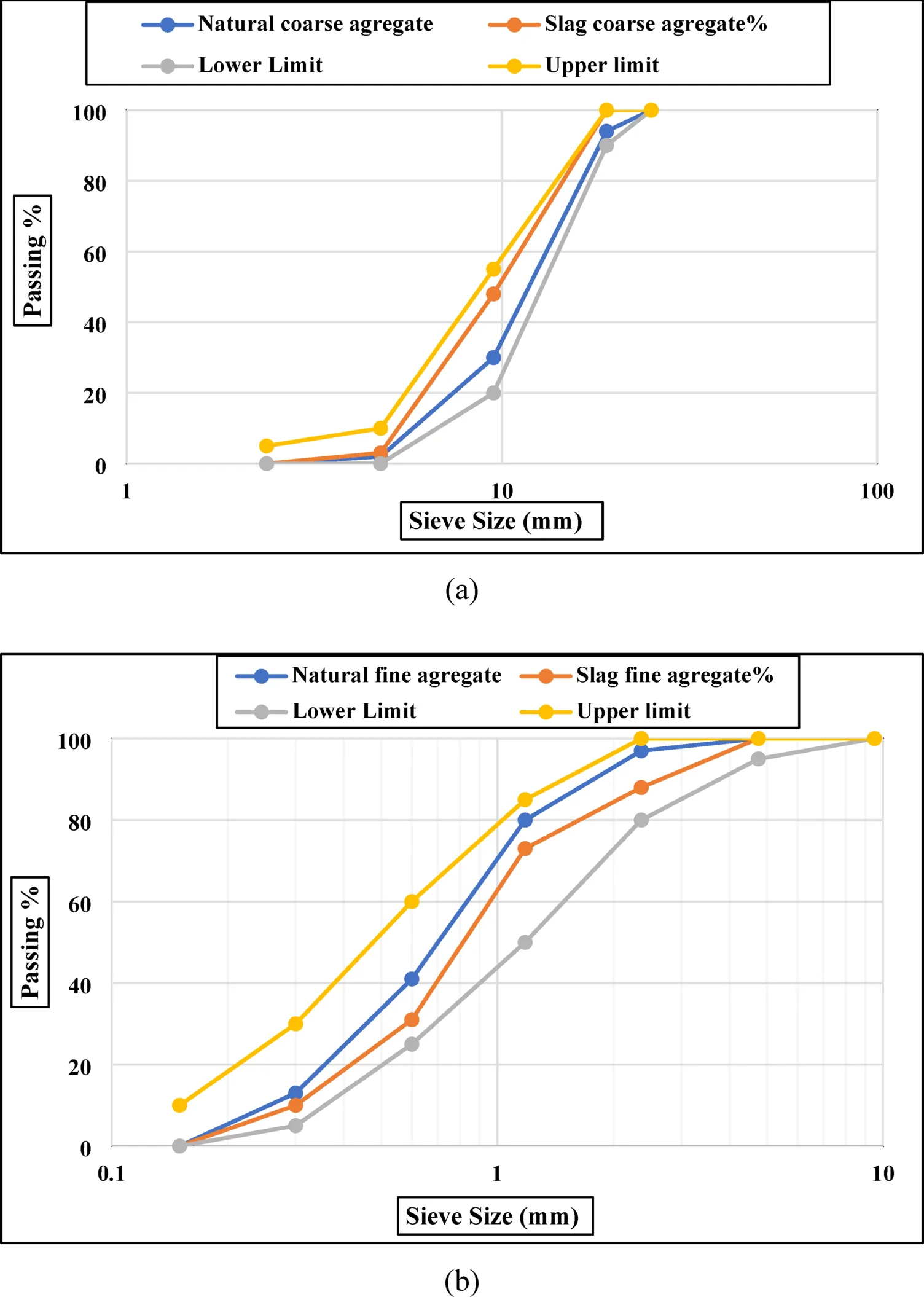
Sieve analysis of the used aggregates: (**a**) coarse aggregate, and (**b**) fine aggregate.

To evaluate the structural potential of slag as a sustainable aggregate, this study replaced 100% of the natural coarse and fine aggregates in the concrete mix with slag aggregates, allowing a comprehensive assessment of mechanical properties, workability, and durability, and providing insight into the feasibility of steel slag as a full aggregate replacement in reinforced concrete slabs. Figure 1 and (Tables 6 and 7) presents the sieve analysis per the Egyptian Code of Practice for Design and Construction of Concrete Structures (ECP 203) and ASTM C33^47^ limits.

**Table 6:** Grading of natural and slag coarse aggregate compared to ASTM C33 limits.

	Sieve Size (mm)	Natural aggregate (%)	Slag aggregate (%)	Lower Limit (%)	Upper limit (%)
Coarse Aggregate	2.36	0%	0%	0%	5%
	4.75	2%	3%	0%	10%
	9.5	30%	23%	20%	55%
	19	94%	90%	90%	100%
	25	100%	100%	100%	100%

**Table 7:** Grading of natural and slag fine aggregate compared to ASTM C33 limits.

	Sieve Size (mm)	Natural aggregate (%)	Slag aggregate (%)	Lower Limit (%)	Upper limit (%)
Fine Aggregate	0.15	0	0	0	10
	0.3	13	10	5	30
	0.6	41	31	25	60
	1.18	80	73	50	85
	2.36	97	88	80	100
	4.75	100	100	95	100
	9.5	100	100	100	100

In the laboratory, large-sized slag aggregates were reduced in size using a jaw crusher to obtain material suitable for use as coarse and fine aggregates in concrete production, after which the crushed material was manually processed through sieve analysis to separate it into the required size fractions. The main constituents of blast furnace steel slag include limestone (CaO) and silica (SiO₂). Other significant components include alumina (Al₂O₃), magnesium oxide (MgO), and trace amounts of sulfur (S). Understanding the chemical composition of slag is essential to predict its performance and ensure its suitability as a full replacement for natural aggregates in reinforced concrete slabs. As per ASTM standards, slag aggregate was tested for grading^48^, unit weight^49^, specific gravity^50^, absorption capacity^50^, and abrasion resistance^51^, soundness^52^, and free CaO and MgO content. Table 5 shows the values obtained: a bulk density of 1450 kg/m³, a specific gravity of 2.68, a water absorption of 1%, an abrasion resistance (Los Angeles abrasion loss) of 25%, a soundness loss of 0.3%, and free CaO and MgO contents of 2% and 0.5%, respectively — all within the acceptable ranges typically specified for concrete aggregates. The low soundness loss and low free-lime/free-magnesia content indicate minimal potential for volumetric expansion, supporting the long-term dimensional stability of the slag aggregate as a full replacement for natural coarse and fine aggregates.

### Test specimens

**Fig. 2:**
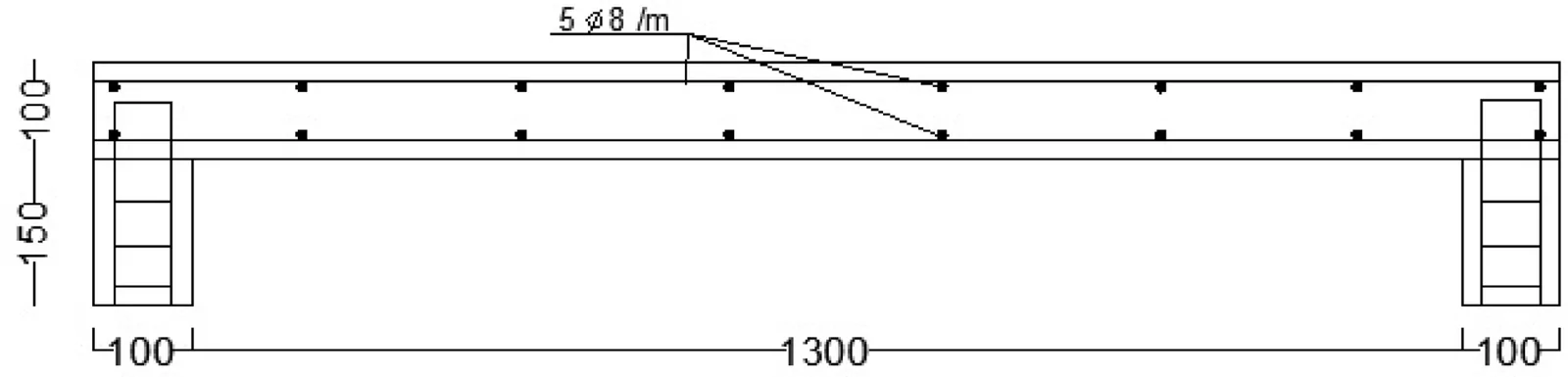
Tested slabs dimensions details, all dimensions are in mm.

**Fig. 3:**
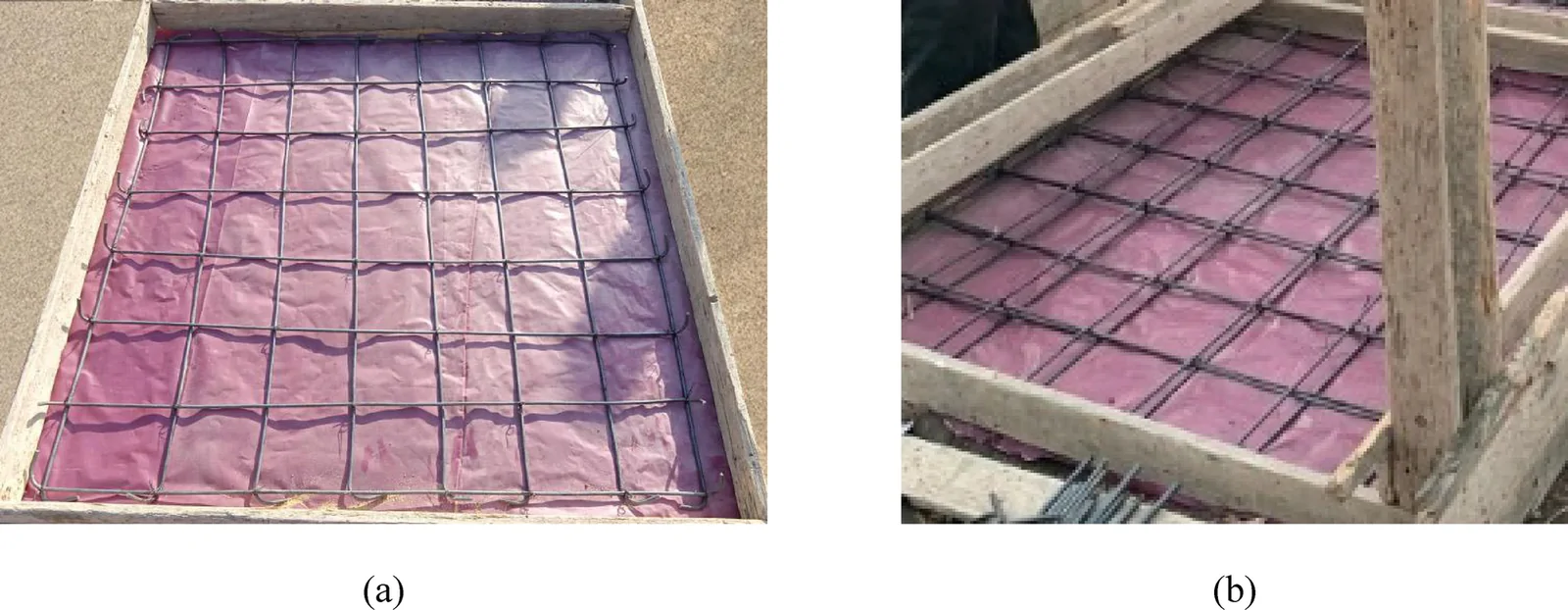
Form description: (**a**) before and (**b**) after putting formwork of stubs columns.

A total of five slabs were tested, each with dimensions of 1500 × 1500 × 100 mm. The slabs were simply supported on four columns located at each corner, with the columns measuring 100 × 100 mm in cross-section and 150 mm in height, as illustrated in shown in Fig. 2; Table 8. Figure 3a shows the slab reinforcement mesh placed inside the formwork before installation of the column stub formwork, and Fig. 3b shows the formwork after placement of the corner column stub forms, clearly showing that the column stub reinforcement is independent of the slab mesh. All tested slabs were reinforced with top and bottom meshes of 5Ø8/m in both directions, providing adequate flexural resistance. The supporting columns were reinforced with vertical high-grade steel bars of Ø 10 mm to carry axial loads, while horizontal mild steel stirrups of Ø 8 mm were provided to ensure confinement and shear resistance. This reinforcement arrangement ensured realistic boundary conditions for evaluating the structural behavior of the slabs under flexural and bending loads. A concrete cover of 20 mm was provided for both the top and bottom reinforcement layers in the unstrengthened control slab, in accordance with ECP 203 requirements for normal exposure conditions. For the strengthened slabs, only the top reinforcement layer was provided with the 20 mm cover, as the bottom face was subsequently strengthened using the corresponding strengthening method. Although the corner column stubs and the slab were cast monolithically in a single pour, the column stub reinforcement acts as compression-only supports with no moment-transferring connection to the slab reinforcement mesh, supporting an approximately simply supported idealization of the boundary conditions of all tested specimens. Although the slabs rest on corner column stubs simulating pinned-support conditions, dual-layer reinforcement (top and bottom) was provided in all specimens to replicate realistic flat slab panel construction practice and to ensure consistent reinforcement configurations across all test specimens. This also prevents premature top-face cracking at the support zones and allows meaningful comparison between strengthening techniques.

**Table 8:** Description, dimensions, and details of tested slabs.

slab no	slab detail				Repair layer	
	slab type	Dimension (mm)	Thickness (mm)	fcu (*N*/mm^2^)	Thickness (mm)	fcu (*N*/mm^2^)
S1	Control slab	1500 × 1500	100	25	-----	-----
S2	Slab with FRP	1500 × 1500	80	25	4	------
S3	Steel fiber concrete	1500 × 1500	80	25	20	33
S4	High-performance concrete	1500 × 1500	80	25	20	35
S5	Slag concrete	1500 × 1500	80	25	20	36

Note: Specimen thicknesses vary by strengthening method to reflect realistic field-representative retrofit scenarios.

The forms used for casting the slabs were constructed from wooden plates to ensure smooth, flat, and parallel surfaces, which are critical for maintaining dimensional accuracy and proper alignment of the reinforcement. The formwork was carefully designed to accommodate the casting of both the slabs and the integrated column bodies, ensuring accurate positioning and monolithic behavior between slabs and supports. This setup allowed for proper compaction and finishing of the concrete, minimizing surface irregularities and potential defects. Detailed specifications and dimensions of the formwork are presented in Fig. 3. Furthermore, Table 8 provides a detailed description of the tested slabs, including their geometric dimensions, reinforcement layout, and the specific strengthening methods applied to each specimen. The five tested specimens were designed to represent a realistic strengthening/retrofit scenario rather than a direct comparison of strengthening materials on an identical as-built section. The control specimen was cast at the full 100 mm slab thickness with no strengthening applied, representing the baseline unstrengthened condition. The remaining four specimens were cast at a reduced base thickness of 80 mm to simulate a deteriorated or deficient slab section (e.g., representative of concrete cover loss or a pre-existing weakened tension zone), which was subsequently strengthened on the tension (bottom) face to restore or enhance its load-carrying capacity. For the FRP-strengthened specimen, the 80 mm base slab was strengthened using an externally bonded FRP sheet approximately 4 mm thick, consistent with standard FRP strengthening practice, where the reinforcing layer itself is thin. For the remaining three strengthening systems — steel fiber-reinforced concrete, high-performance concrete (HPC), and blast furnace steel slag aggregate concrete (the focus of this study) — a 20 mm cast overlay was applied to the 80 mm base slab, bringing the total strengthened section to 100 mm, matching the control slab depth and providing a consistent basis for comparison at equal overall thickness.

The resulting difference in strengthening-layer thickness between the FRP specimen (4 mm) and the three overlay-based specimens (20 mm) is an inherent consequence of the fundamentally different strengthening mechanisms involved — an externally bonded thin composite layer versus a cast cementitious overlay — rather than an inconsistency in experimental design.

### Mixing and casting

The coarse and fine aggregates were first dry-mixed for 2–3 min to achieve a homogeneous distribution. Subsequently, cement was added and mixed for an additional 1–2 min. Approximately 80% of the mixing water was added initially, followed by the remaining 20% after 2–3 min, and the mixing continued for 3 more minutes to ensure uniformity of the concrete mixture. Prior to casting, the wooden moulds were thoroughly cleaned, and the reinforcement cages were carefully positioned to maintain accurate placement and cover. After the curing of the initial concrete layer for 28 days, the strengthening layers were applied using various techniques according to the experimental plan. Prior to bonding-agent application, the substrate surface was roughened using a wire brush to remove surface laitance and loose material, followed by cleaning with compressed air to remove dust and debris. Kima Boxy 104 bonding agent — an epoxy-based adhesive — was then applied to the clean, dry substrate immediately before casting the overlay, in accordance with the manufacturer’s application guidelines, to improve adhesion between the new strengthening layer and the existing concrete surface. Mechanical vibration was applied to ensure proper compaction and eliminate voids, promoting a monolithic bond between the original slab and the strengthening layer.

### Test setup

**Fig. 4:**
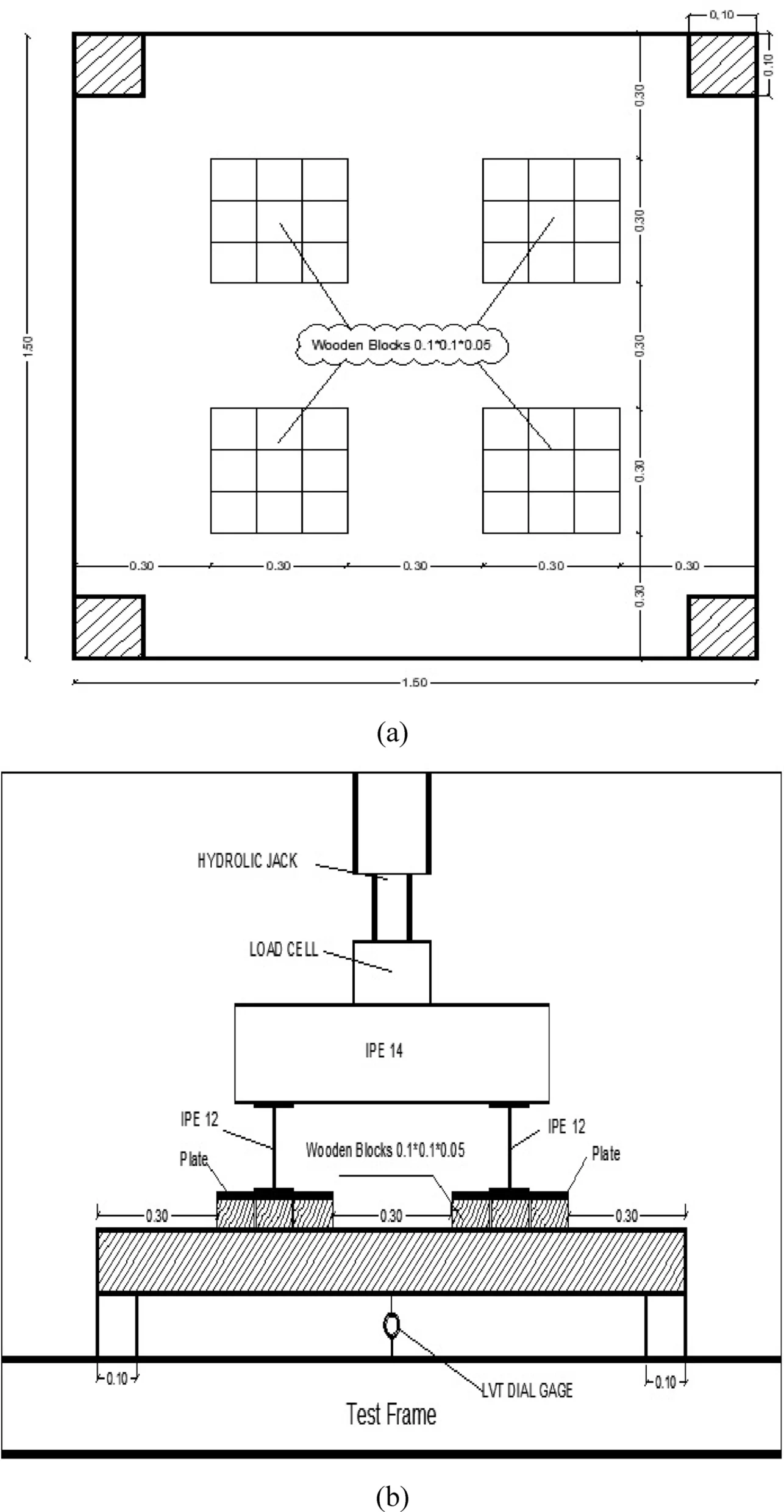
Layout of instruments and test setup: (**a**) plan of tested slab and (**b**) tested slab elevation.

All slab specimens were tested under concentric static loading using a 1000 kN hydraulic jack mounted on a robust steel frame within the RC laboratory. The slabs were carefully positioned on a supporting frame comprising three B.F.I. No. 20s and B.F.I. No. 12 beams resting on the frame. The hydraulic jack applied force through a system of steel members arranged in a pyramidal configuration. The bases of the steel I-beams rested on wooden blocks, which helped to distribute the load evenly across the slab surface and prevent localized stress concentrations. This setup minimized eccentricity and uneven loading, enabling precise and reliable measurement of the slabs’ flexural response, as the four-point loading configuration (Fig. 4) was specifically designed to induce a predominantly flexural stress state in the central region of the slab, free of significant shear effects. one LVDT with 100 mm was used and located on the top side of the specimen This arrangement ensured that the experimental results accurately reflected the performance of each slab and allowed for meaningful comparison between different strengthening techniques.

### Numerical simulation using the finite element (FE) method

Numerical simulation provides a powerful and cost-effective tool for investigating structural behavior, offering rapid insights compared to experimental testing. To ensure accuracy and reliability, it is essential to validate finite element (FE) models against experimental results. In this study, a three-dimensional nonlinear FE model was developed using ANSYS version 12 to simulate the behavior of reinforced concrete (RC) slabs under various strengthening configurations. The model was designed to predict key structural responses, including displacements, strain distributions, stress contours, shear forces, failure modes, and crack propagation patterns. The nonlinear behavior of both concrete and reinforcing steel was incorporated into the model. Concrete was represented using SOLID65 elements, which are capable of simulating tensile cracking as well as compression crushing, providing realistic modeling of RC behavior under flexural and shear loads. To control post-cracking shear stiffness, shear transfer coefficients were assigned values of 0.3 for open cracks and 1 for closed cracks, following established modeling practices. Reinforcing steel, including both main longitudinal bars and web reinforcement, was modeled using LINK180 elements embedded within the concrete mesh defined by the SOLID65 elements. Self-weight of the slab and overlay was inherently accounted for in the ANSYS model through the assigned material density of each element in conjunction with the model’s gravitational acceleration (ACEL) setting, rather than as a separately defined manual load step. This approach allows for accurate interaction between steel and concrete, reflecting the composite action of reinforced slabs. The modeling strategy and element selection were adopted based on validated approaches from previous studies^53–57^ ensuring that the FE simulations reliably replicate the observed experimental behavior of the tested slabs.

**Fig. 5:**
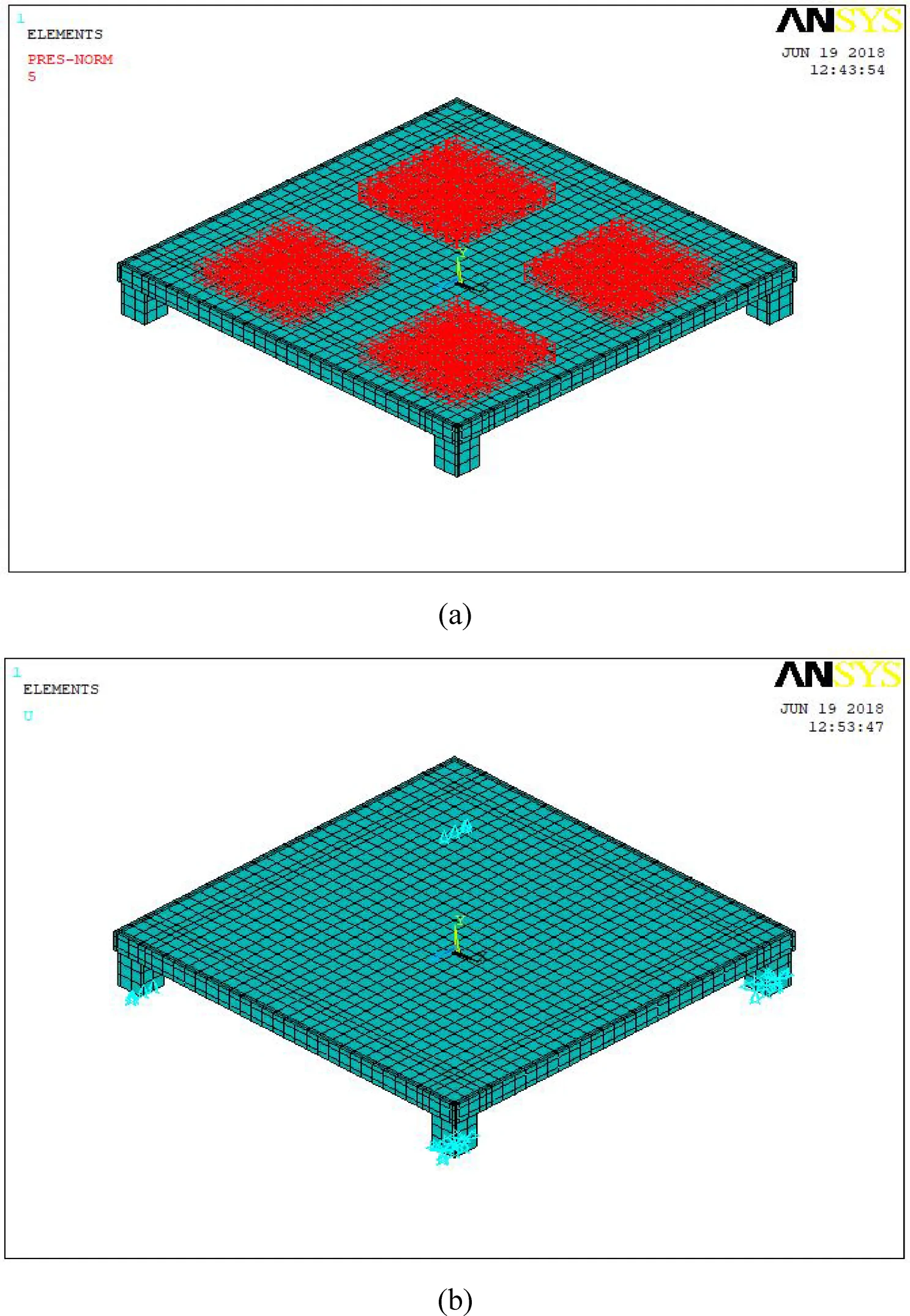
Geometric modeling of reinforced concrete slabs in ANSYS: (**a**) load distribution on the slab surface and (**b**) slab supports.

After defining the model geometry with volumes, areas, lines, and key points, the model was meshed into small finite elements to enable accurate finite element analysis (FEA). Displacement-controlled loading was applied to four designated pressure load areas, with the total displacement divided into multiple incremental steps to capture the nonlinear behavior of the slabs. The Newton-Raphson iterative method was employed to ensure convergence of the solution within specified tolerance limits, providing stable and reliable numerical results. Additionally, ANSYS’s automatic time-stepping algorithm was utilized to adaptively adjust load step sizes, improving computational efficiency while maintaining accuracy in capturing nonlinear responses such as cracking, yielding of reinforcement, and progressive failure mechanisms. Figure 5 show the slab supports, load distribution, and the finite element model.

### Parametric study

A comprehensive numerical investigation was conducted to examine variables beyond the scope of the experimental program through three-dimensional finite element modeling in ANSYS. Twenty-two slab models with 1500 × 1500 mm were simulated to evaluate structural behavior under varying strengthening configurations and loading conditions. The parametric study systematically investigated three primary variables: (1) strengthening layer thickness, comparing the performance of slag concrete versus conventional concrete overlays; (2) slab rectangularity ratio, analyzing the influence of aspect ratio variations while maintaining constant strengthening layer thickness, in which slab dimensions were varied from 1500 × 1500 mm to 3000 × 1500 mm by increasing slab length while keeping slab width fixed at 1500 mm; and (3) reinforcement corrosion in the bottom steel mesh of the strengthening layer, assessing its impact on flexural response. Reinforcement corrosion is modeled as a uniform reduction in bar diameter, consistent with approaches used in previous numerical studies of corroded RC members^58^. This approach captures the primary structural effect of corrosion (reduction in tensile resistance) but does not account for: (a) bond degradation between corroded bars and surrounding concrete, (b) non-uniform pitting corrosion patterns, or (c) the expansion pressure exerted by corrosion products causing concrete cover cracking. These effects are acknowledged as limitations of the current model. Future studies should incorporate interface elements with degraded bond properties to capture these secondary corrosion effects. The complete matrix of parametric configurations and corresponding model specifications is presented in Table 9, establishing a systematic framework for quantifying the influence of these critical design parameters on structural performance.

**Table 9:** Studied slabs for repair layer thickness.

Parametric study	Slab number	Main slab				Repair layer	
		Slab Dim (mm)	Slab RFT	Thickness (mm)	Fcu	Thickness (mm)	Fcu
			TOP&BOT		(N/mm^2^)		(N/mm^2^)
Repair thickness using slag concrete	S1	1500 × 1500	5 Ø 8 /m	100	25		
	S2	1500 × 1500	5 Ø 8 /m	80	25	0	0
	S3	1500 × 1500	5 Ø 8 /m	80	25	20	36
	S4	1500 × 1500	5 Ø 8 /m	80	25	30	36
	S5	1500 × 1500	5 Ø 8 /m	80	25	40	36
	S6	1500 × 1500	5 Ø 8 /m	80	25	50	36
Repair thickness using ordinary concrete	S1	1500 × 1500	5 Ø 8 /m	100	25		
	S2	1500 × 1500	5 Ø 8 /m	80	25	0	0
	S3	1500 × 1500	5 Ø 8 /m	80	25	20	25
	S4	1500 × 1500	5 Ø 8 /m	80	25	30	25
	S5	1500 × 1500	5 Ø 8 /m	80	25	40	25
	S6	1500 × 1500	5 Ø 8 /m	80	25	50	25
Losses in bottom steel mesh	S1	1500 × 1500	5 Ø 8 /m	80	25	20	36
	S2	1500 × 1500	5 Ø 6 /m	80	25	20	36
	S3	1500 × 1500	5 Ø 4 /m	80	25	20	36
	S4	1500 × 1500	5 Ø 2 /m	80	25	20	36
	S5	1500 × 1500	0	80	25	20	36
Rectangular ratio for slab	S1	1500 × 1500	5 Ø 8 /m	80	25	20	36
	S2	1800 × 1500	5 Ø 8 /m	80	25	20	36
	S3	2100 × 1500	5 Ø 8 /m	80	25	20	36
	S4	2400 × 1500	5 Ø 8 /m	80	25	20	36
	S5	2700 × 1500	5 Ø 8 /m	80	25	20	36
	S6	3000 × 1500	5 Ø 8 /m	80	25	20	36

The control specimen S1 (100 mm slab, no repair layer, fcu = 25 N/mm²) is common to both the ‘Repair Thickness Using Slag Concrete’ and ‘Repair Thickness Using Ordinary Concrete’ sub-studies and is counted once. The parametric matrix therefore comprises 22 unique numerical models in total (6 + 6 + 5 + 6 = 23, minus the shared S1 control = 22), consistent with the 22 models.

## Results and discussion

All slab specimens were tested until failure to fully capture their structural behavior under ultimate loading conditions. load and displacement data were recorded continuously using a data acquisition system connected to the test machine, ensuring precise measurement of the slabs’ response throughout the loading process. The experimental results of the strengthened slabs are presented and discussed in the following sections. key performance indicators, including ultimate load, ultimate deflection, crack patterns and failure modes, stiffness, energy dissipation, and toughness, were systematically compared to evaluate the effectiveness of the different strengthening techniques. The comparative analysis provides insight into how each strengthening method enhances the structural performance of reinforced concrete slabs, as summarized in Table 10. The performance indicators in Tables 10 and 11 were computed as follows: Initial post-cracking stiffness (kN/mm) = slope of the load-deflection curve between the first crack load and the load corresponding to 75% of the ultimate load. Energy dissipation (kN·mm) is defined as the area under the load–deflection curve from zero up to the ultimate load, approximated as the product of the ultimate load and the corresponding deflection (Pu × δu). Toughness (kN·mm) is defined as the total area under the load–deflection curve from zero up to the ultimate deflection at failure, computed using the trapezoidal numerical integration rule applied to the recorded load–deflection data, and therefore includes the post-peak descending branch of the response where applicable.

**Table 10:** Results of test specimens.

Slab No	Descreption	Frist Crack Load (kN)	Ultimate Load (kN)	Ultimate Deflection (mm)	Initial Stifness (kN/mm)	Energy Dissipation (kN.mm)	Toughness (kN.mm)
S_1_	Control Slab	51	164	48.2	4.1	7905	2760
S_2_	Slab With FRP	41	241	18.6	15.5	4483	3108
S_3_	Steel Fiber Concrete	60	234	23.8	11.8	5569	3410
S_4_	High Performance Concrete	62	195	19	12.3	3705	2950
S_5_	Slag Concrete	59	213	21	12.2	4473	3365

**Table 11:** Experimental results of test specimens.

specimens	Ultimate load Pu (KN)	1st cracking load (KN)	Ultimate Deflection δu (mm)	Toughness (KN.mm)	Post cracking Stiffness (kN/mm)
S_1_	164	51	48.2	2760	4.1
S_2_	241	41	18.6	3108	15.5
S_3_	234	60	23.8	3410	11.8
S_4_	195	62	19.0	2950	12.3
S_5_	213	59	21.0	3365	12.2

### Load-displacement relationship for slabs

**Fig. 6:**
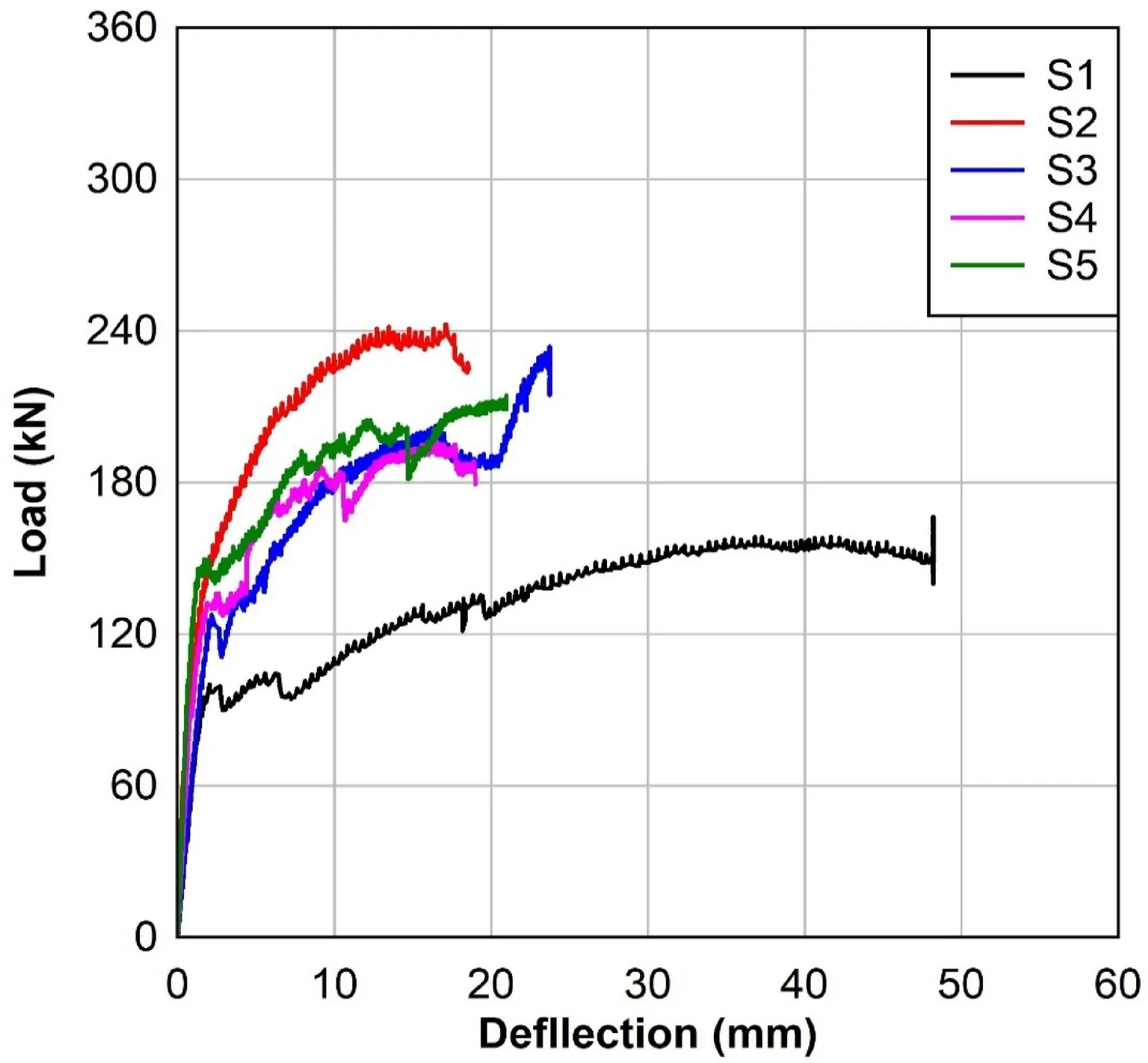
Load – flexure displacement for tested slabs.

Figure 6 shows a comparison between the applied loads and the corresponding central deflection curves of the tested slabs. The results indicate that the strengthened slabs exhibited improved load-carrying behavior compared to the control slab. Specifically, the deflection and ultimate load data demonstrate that the application of strengthening layers led to a noticeable increase in the ultimate load for the same deflection level as the control slab. The load-deflection curves reveal three distinct behavioral stages for all specimens: a linear elastic pre-cracking stage characterized by steep initial stiffness, a post-cracking nonlinear stage with progressive stiffness degradation as cracks widened and propagated, and a final yielding-to-failure stage marked by a plateau or gradual load drop toward ultimate failure. The control slab S1 exhibited the most gradual stiffness degradation with the largest ultimate deflection of 48.2 mm, reflecting a more ductile response, whereas the strengthened slabs S2–S5 showed steeper post-cracking stiffness but reached their ultimate loads at significantly smaller deflections ranging from 18.6 mm to 23.8 mm. In terms of ductility, the control slab demonstrated superior deformation capacity compared to the strengthened slabs, which is attributed to the increased stiffness introduced by the strengthening materials that limited deflection while enhancing load resistance. Notably, S5-slag concrete achieved a balanced response, combining a 29.9% increase in ultimate load with a moderate ultimate deflection of 21.0 mm, indicating an acceptable level of ductility alongside improved strength. These observations confirm that while all strengthening techniques effectively enhanced load capacity, the slag concrete overlay offered a more favorable balance between strength gain and ductile deformation behavior.

### Ultimate load, first cracking loads, toughness, and post-cracking stiffness

The experimental results of the tested slabs, including ultimate loads, first cracking loads, ultimate deflection, toughness, and post-cracking stiffness, are summarized in Table 11. The ultimate load capacity increased by approximately 47% (S2-FRP), 42.7% (S3-steel fiber), 18.9% (S4-HPC), and 29.9% (S5-slag concrete) compared to the control slab S1. These results are in good agreement with the trend reported in previous studies, where the ultimate load capacity of beams incorporating different ratios of concrete slag increased by 122.4% to 146.5% compared to a reference beam^36^. The results also indicate that the first cracking loads of the strengthened slabs were generally lower than those of the control slab, suggesting earlier crack initiation due to the modified material or reinforcement configuration. Among all specimens, S2-FRP recorded the lowest first cracking load of 41 kN, reflecting a 19.6% reduction compared to the control slab S1 (51 kN). On the other hand, slabs S3, S4, and S5 demonstrated higher first cracking loads, with S5-slag concrete recording 59 kN, representing an increase of 15.7% over the control slab (Table 12). This improvement indicates that the incorporation of slag concrete delayed the onset of cracking, likely due to its enhanced tensile resistance and pore refinement characteristics that contribute to a denser and more crack-resistant matrix. This behavior can be further interpreted through the underlying mechanisms of steel-slag aggregate concrete: the angularity and rough surface texture of the steel-slag aggregate particles improve mechanical interlock with the surrounding cement paste, the interfacial transition zone (ITZ) is likely densified relative to natural-aggregate concrete, and these effects together promote more effective crack-bridging and stress-transfer across the crack faces, delaying crack initiation and slowing crack propagation. This interpretation is consistent with the mechanisms reported in the existing literature on steel slag aggregate concrete^36^. These findings are consistent with the trend observed in previous studies, where the first cracking load capacity of slag concrete beams increased significantly by 172.4% for beam, respectively, compared to the reference beam^36^. However, the differences in ultimate deflection and post-cracking stiffness are substantial rather than small: the strengthened slabs exhibit 50–60% reductions in ultimate deflection and approximately 190–280% increases in post-cracking stiffness relative to the control slab, alongside a 30–53% reduction in energy dissipation and a comparatively modest 7–24% increase in toughness. These findings indicate that the strengthening techniques substantially increased stiffness and reduced deflection and energy dissipation, while producing a more modest change in toughness. These findings provide valuable insight into the efficiency and effectiveness of different strengthening methods for reinforced concrete slabs.

**Table 12:** Summary table of percentage changes (relative to control slab S1):

Specimen	1st Crack Load	Ultimate Load	Ultimate Deflection	Post-Cracking Stiffness	Energy Dissipation	Toughness
S2 – FRP	−19.6%	+ 47.0%	−61.4%	+ 278.0%	−43.3%	+ 12.6%
S3 – Steel Fiber Concrete	+ 17.6%	+ 42.7%	−50.6%	+ 187.8%	−29.6%	+ 23.6%
S4 – HPC	+ 21.6%	+ 18.9%	−60.6%	+ 200.0%	−53.1%	+ 6.9%
S5 – Slag Concrete	+ 15.7%	+ 29.9%	−56.4%	+ 197.6%	−43.4%	+ 21.9%

### Comparison between the experimental and analytical results

**Fig. 7:**
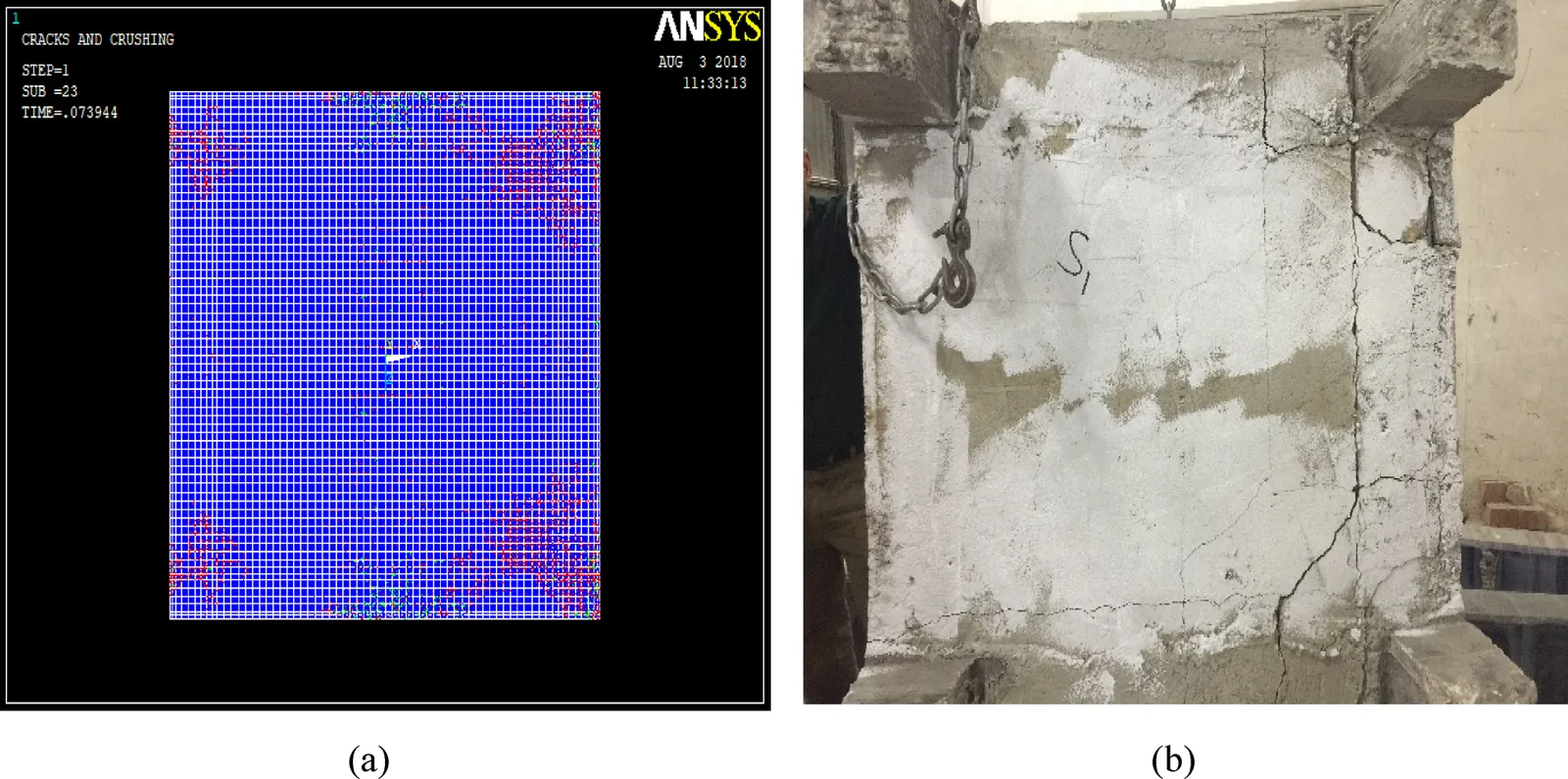
Tested slabs following failure. (**a**) crack patterns for F.E.M and (**b**) crack patterns for EXP.

All tested slab specimens failed through a combination of flexural and punching shear cracking, as documented by the crack patterns recorded at each load increment. The crack patterns for EXP and F.E.M. tested slabs following failure is shown in Fig. 7. The tested slabs were subjected to a quasi-uniform load on its upper surface, resulting in flexural cracks on the bottom surface at the center, shear cracks inclined near the corners, and fine compressive cracks on the top surface. In the F.E.M analysis, the stress distribution showed tensile stresses concentrated at the bottom and compressive stresses at the top, with a deflection shape and maximum displacement at the center. the locations of the actual cracks matched the numerical analysis results, confirming its accuracy in representing the structural flexural behavior.

**Fig. 8:**
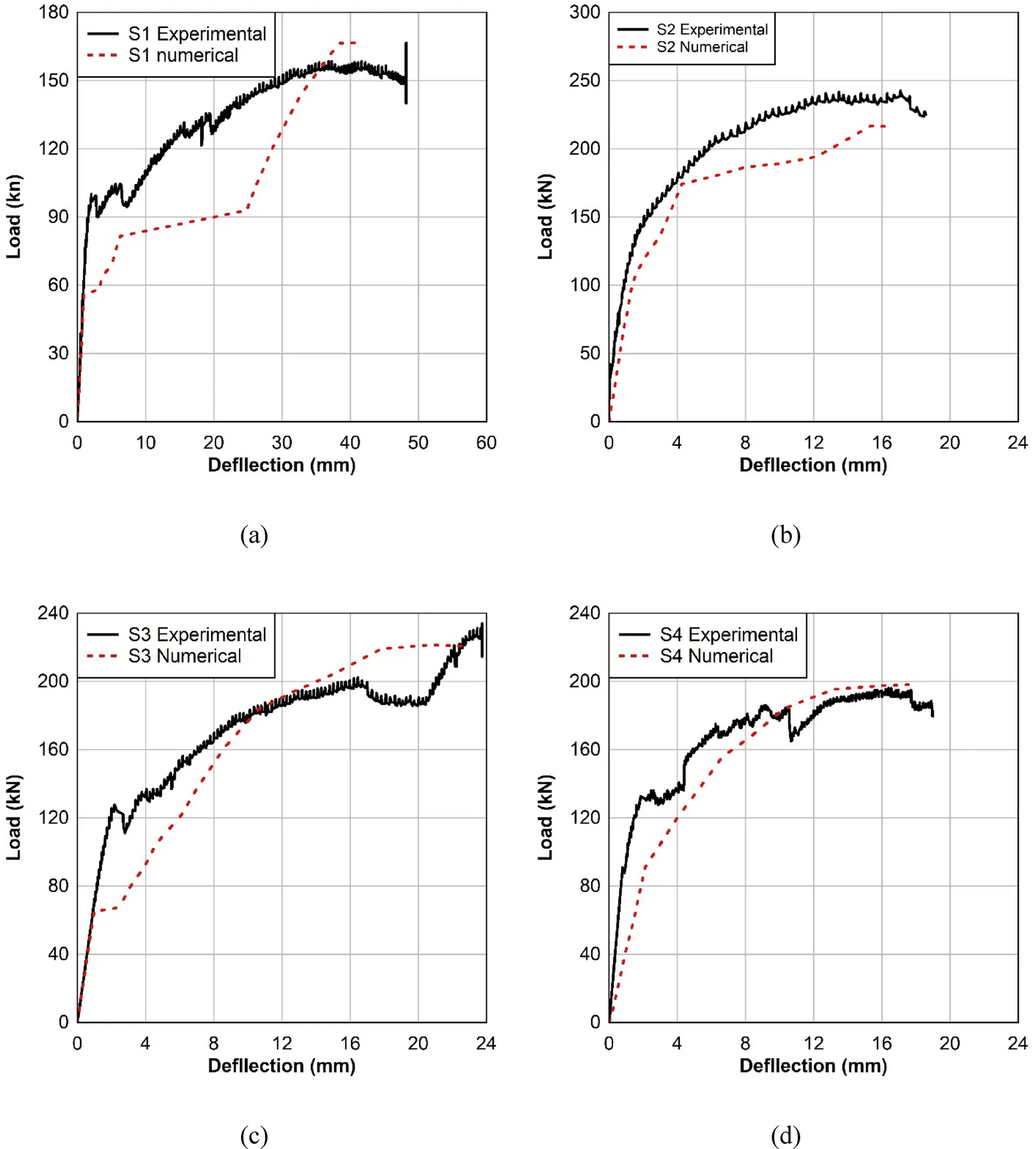

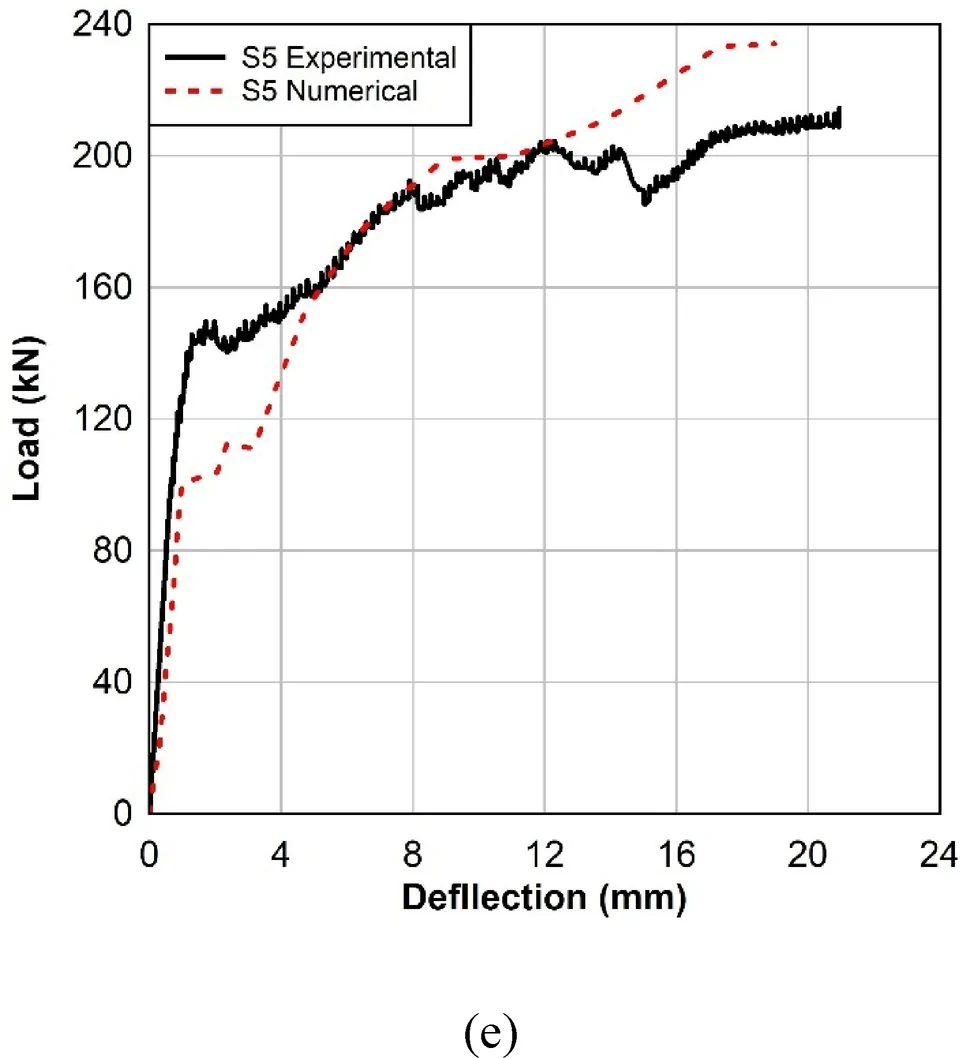
Comparison between experimental and finite element results. (**a**) experimental/numerical load–deflection for S_1_, (**b**) experimental/numerical load–deflection for S_2_, (**c**) experimental/numerical load–deflection for S_3_, (**d**) experimental/numerical load–deflection for S_4_ and (**e**) experimental/numerical load–deflection for S_5_.

Figure 8 investigate a comparison between the load-deflection relationship for experimental and numerical results. The ultimate load and deflection for both experimental and FE results are summarized in Table 13. The average percentage between experimental results and numerical results in the ultimate load is 94%, while it is 91% for ultimate deflection. It is clarified that these values represent the ratio of the experimental to the numerical result (i.e., experimental/numerical × 100%) for each corresponding quantity, rather than a normalized prediction error. Moreover, it can be seen that the numerical results average difference of 6% and 9%with the experimental ones, and the finite element method is representing the best tested slabs. It should be noted that nearest agreement is quantitative for ultimate load and deflection (94% and 91%, respectively) and qualitative for crack pattern and failure mode, based on the visual comparison between experimental and numerical crack patterns shown in Fig. 7. This close correlation confirms the reliability of the numerical model, which was subsequently employed to conduct the 22-configuration parametric study, thereby extending the validated experimental dataset — limited to a single specimen per configuration — to a broader range of geometric and material parameters. Mesh sensitivity was evaluated using three element sizes — coarse (50 mm), medium (25 mm), and fine (15 mm). The medium mesh (25 mm) demonstrated satisfactory convergence, producing results within 2% of the fine mesh solution while reducing computational time by approximately 60%, and was therefore adopted for all models. The force convergence criterion was set to 0.5% (0.005), which is the ANSYS program default value, ensuring numerical stability and solution accuracy throughout all analyses. This is consistent with established numerical simulation practice, whereby simulations are controlled through user-defined convergence criteria related to the evolving model state, such as displacement thresholds or force convergence^59,60^. The Newton-Raphson iterative scheme was employed to ensure solution convergence at each load increment. The key model assumptions include: (1) perfect bond between steel reinforcement and concrete (no slip elements); (2) concrete tensile behavior captured through the smeared crack model in SOLID65 with a modulus of rupture of 0.62√fcu (MPa); and (3) exclusion of shrinkage and creep effects, which is a standard simplification in short-term static loading simulations. It is emphasized that the perfect-bond assumption between reinforcement and concrete does not capture bond-slip behavior, and, consistent with the corrosion-modeling limitations already noted in Sect. 2.6, does not represent bond degradation, non-uniform pitting corrosion, or corrosion-induced cover cracking; these remain acknowledged simplifications of the present model. Despite these simplifications, the average accuracy of 94% for ultimate load and 91% for deflection across all five tested specimens confirms the reliability of the model for the purposes of this parametric study.

**Table 13:** Ultimate load and deflection for both experimental and FE results.

Specimens	Experimental		Numerical		Exp to Num%	
	Ultimate load (kN)	Deflection (mm)	Ultimate Load (kN)	Deflection (mm)	Load (kN)	Deflection (mm)
S_1_	164.0	48.2	166.5	41.7	98.5	86.5
S_2_	240.8	18.6	216	16.7	88.5	89.8
S_3_	234.0	23.8	220.5	22.8	94.0	95.8
S_4_	194.5	19.0	198	17.6	98.2	92.6
S_5_	213.0	21.0	234	19.0	91.0	90.5
Average					94.0	91.0

The rotation observed at the corner column stubs in the numerical deflection contour (Fig. 8a) is attributed to the displacement-controlled loading protocol in ANSYS, which continues to apply incremental displacement steps beyond the experimental failure point to capture the full post-peak response. This post-failure behavior causes additional column stub rotation in the numerical model that is not observed experimentally, where loading was terminated at slab failure. The pre-failure numerical response with the experimental results, as confirmed by the 94% and 91% average accuracy for ultimate load and deflection respectively. The nonlinear numerical analysis shows close behavior with those obtained from the experimental program. The comparison between the experimental results with the finite element study shows that both models are in good accuracy with each other and shows the compatibility between the two solutions. Furthermore, the developed finite element method can be used successfully to extend the work for studying the Strengthening of flat concrete slabs with blast furnace slag.

### Parametric study results

**Fig. 9:**
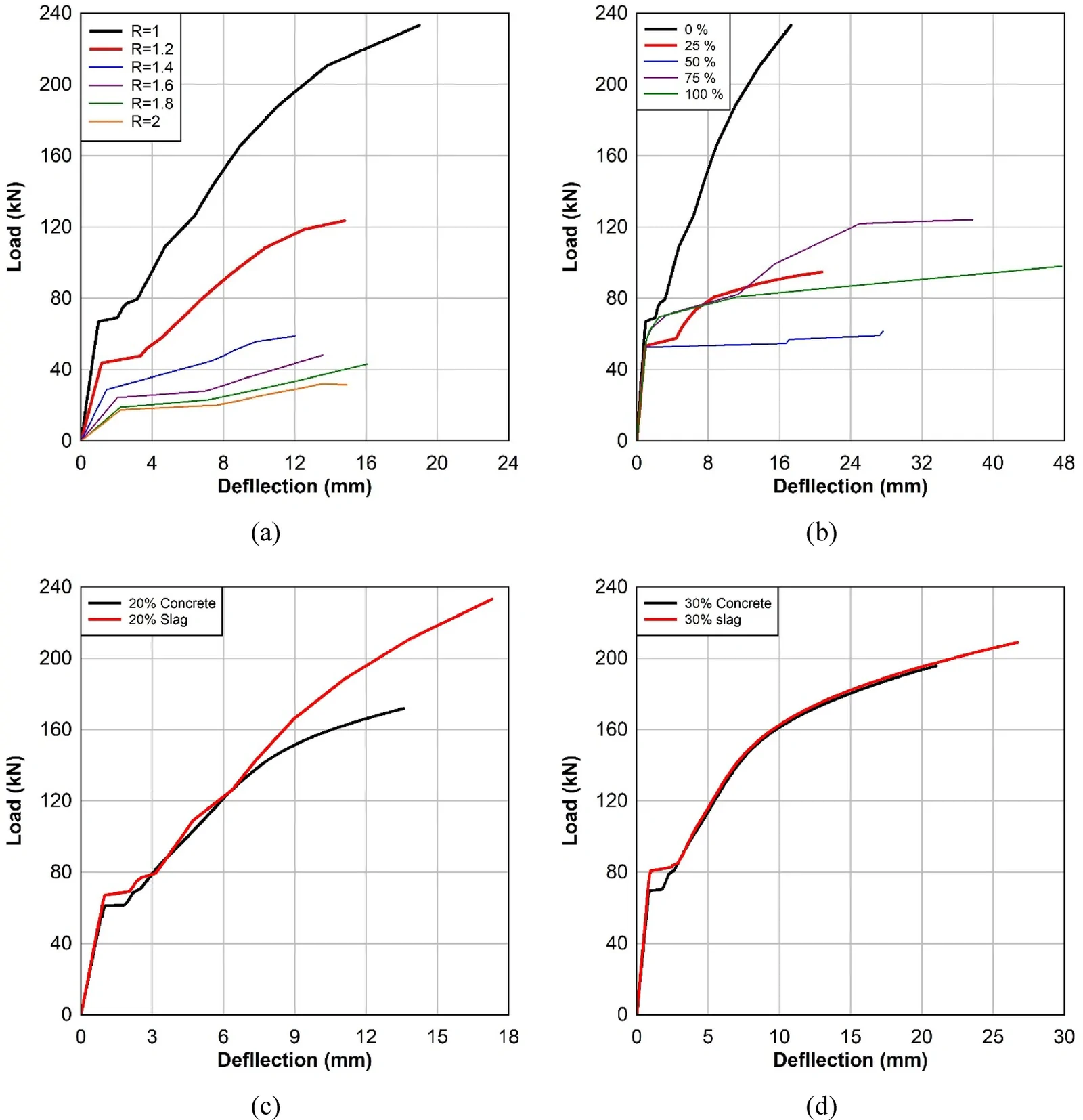

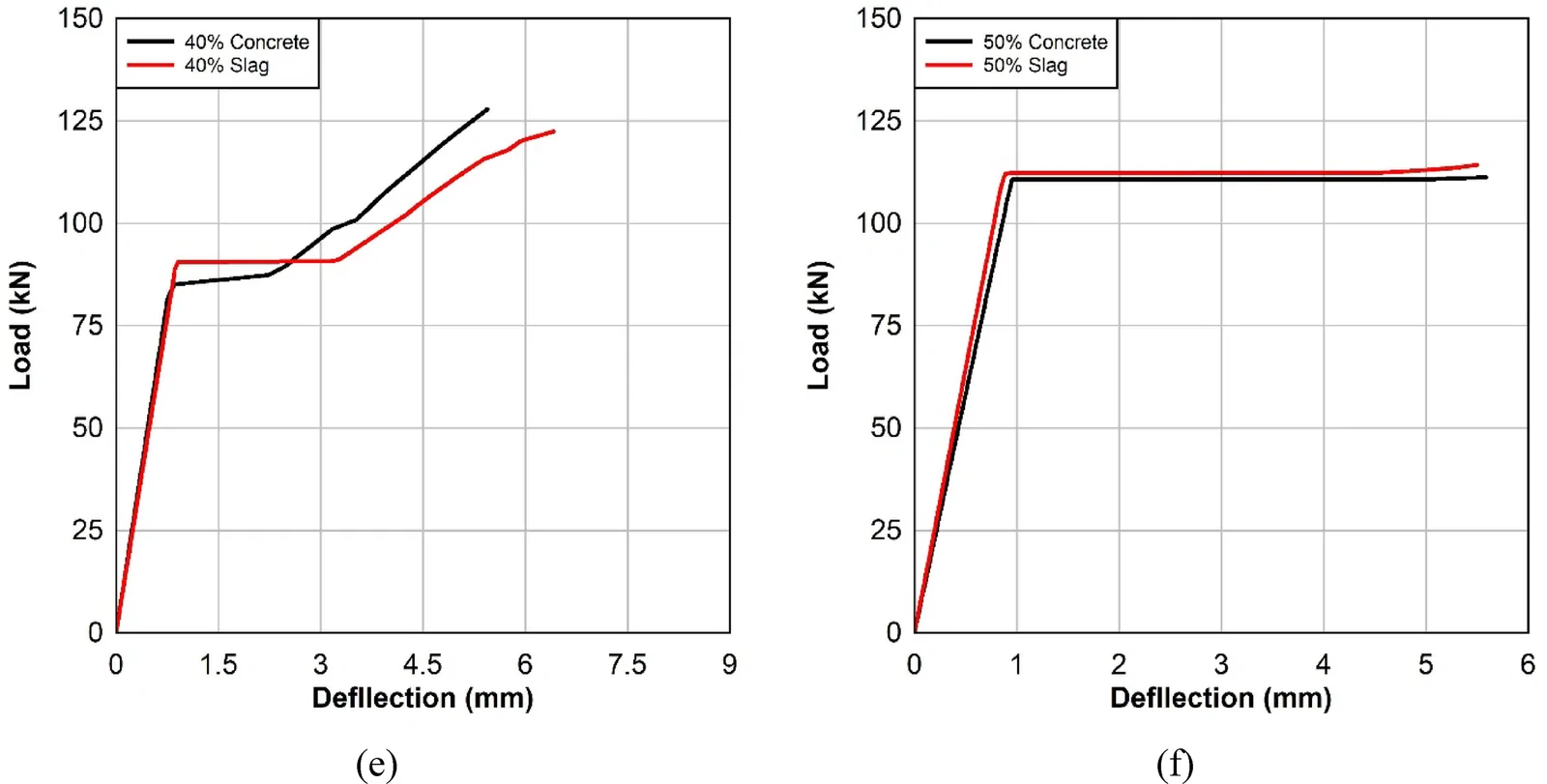
parametric study results: (**a**) load–deflection curve for due to rectangular ratio, (**b**). load–deflection curve for losses in steel mesh, (**c**) 20% repair layer thickness, (**d**) 30% repair layer thickness, (**e**) 40% repair layer thickness and (**f**) 50% repair layer thickness.

Figure 9 presents the load–deflection responses at the mid-span for all numerical models. A detailed summary of the numerical results is provided in Table 14. The results indicate that the ultimate load capacity increases as the repair (overlay) layer thickness increases, up to an optimum of approximately 20–30% of the slab thickness, beyond which the additional self-weight begins to offset the strength gain. Conversely, increasing the rectangularity ratio of the tested slabs from 1.2 to 2.0 leads to a noticeable reduction in ultimate shear capacity due to the increased span and corresponding reduction in effective shear resistance. Table 14 summarizes the ultimate loads, first-crack loads, ultimate and yield deflections, toughness values, and post-cracking stiffness for the proposed numerical models. Moreover, the findings reveal that increasing the loss in the bottom diameter from 25% to 100% results in a substantial reduction in ultimate load capacity, decreasing from 233 kN to 61.5 kN, corresponding to a reduction percentage that increases from 0% (intact) to 73.6% (100% mesh loss). This reduction is also reflected in the first-crack load, ultimate and yield deflection, toughness, and post-cracking stiffness, confirming the critical role of bottom reinforcement in controlling both strength and ductility. In addition, the results show that slabs strengthened with slag concrete exhibit superior performance compared to those strengthened with ordinary concrete, achieving an improvement of approximately 35% in ultimate shear strength and overall post-cracking behavior. However, increasing the thickness of the strengthening layer to more than 30% of the slab’s original thickness leads to a reduction in ultimate shear strength, first-crack load, ultimate and yield deflection, toughness, and post-cracking stiffness. This behavior may be attributed to increased self-weight and altered stress distribution, which negatively affects the slab’s overall shear performance.

**Table 14:** Summary of finite element results.

Study case	Slab specimens’ description	Ultimate load Pu (kN)	1st cracking load (kN)	Ultimate Deflection δu (mm)	Toughness (kN.mm)	Post cracking Stiffness (kN/mm)
rectangularity	*R* = 1	233.3	67.5	17.3	3001	16.2
	*R* = 1.2	123	40.5	14.8	1203	10.0
	*R* = 1.4	59	38.3	12.0	490	5.9
	*R* = 1.6	48.1	27.7	13.6	405	4.3
	*R* = 1.8	43.0	23.1	16.1	416	3.2
	*R* = 2.0	31.5	22.2	14.9	413	2.5
Loses in diameter loss	0% losses	233.3	63.7	17.3	3001	16.2
	25% losses	124.0	53.3	20.8	4042	5.5
	50% losses	98.02	53.1	27.6	3781	4.0
	75% losses	94.7	52.4	37.5	1545	2.7
	100% losses	61.5	50.1	47.7	1503	2.5
Repairing Layer Thickness Using Ordinary Concrete	Control slab	166.4	51.8	45.7	3800	4.4
	No repair layer (Tr = 0%)	140.8	42.8	38.3	1420	3.7
	Tr = 20% Ts	171.9	58.5	13.6	3087	15.2
	Tr = 30% Ts	195.8	67.5	21	1625	11.2
	Tr = 40% Ts	127.8	81	5.5	500	28.1
	Tr = 50% Ts	111.3	119.3	5.6	565	23.9
Repairing Layer Thickness Using Slag Concrete	Control slab	150.1	65.3	63.2	6763	2.9
	No repair layer (Tr = 0%)	140.8	42.8	45.7	1420	3.7
	Tr = 20% Ts	232.2	67.5	17.3	4294	16.2
	Tr = 30% Ts	208.9	78.8	26.7	2605	9.4
	Tr = 40% Ts	135.9	106.4	7.5	736	21.8
	Tr = 50% Ts	135.0	114.3	5.5	570	24.9

### Cracking pattern and mode of failure

**Fig. 10:**
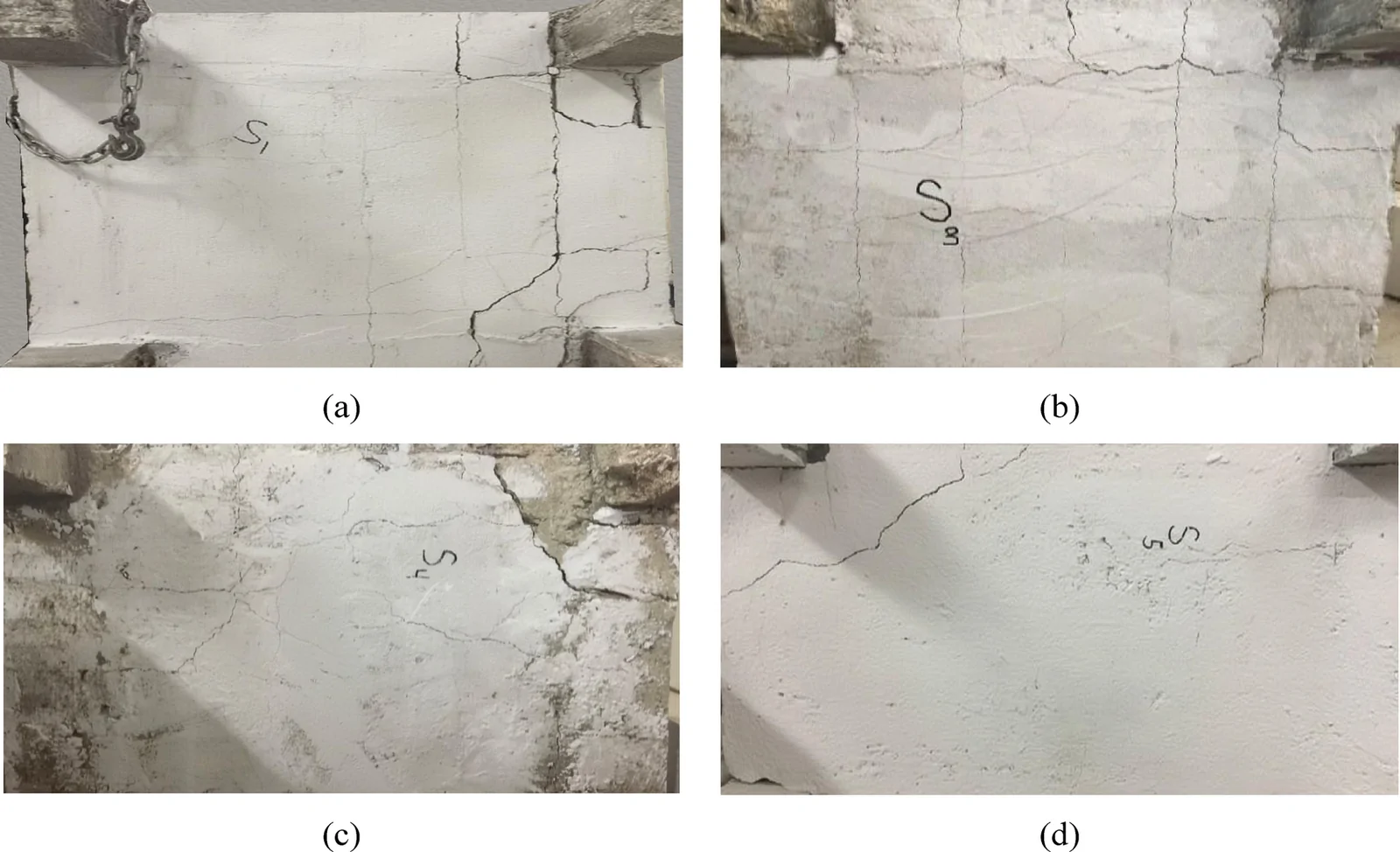
(**a**) crack pattern in tested slab S1, (**b**) crack pattern in tested slab S_3_, (**c**) crack pattern in tested slab S_4_ and (**d**) crack pattern in tested slab S_5_.

Figure 10 illustrates the observed cracking patterns for all tested slabs, recorded in detail upon completion of the experimental program. It was consistently noted that all slabs predominantly failed in shear, underscoring the governing influence of shear resistance in their structural behavior. The dominance of shear-related failure modes in this study is explained by the loading and support configuration. The 1500 × 1500 mm slabs are supported on 100 × 100 mm corner column stubs, creating a large shear span-to-depth ratio that promotes diagonal tension cracking from the loaded region toward the support. Although the load is applied to induce flexure, the combination of the relatively thin slab (100 mm total), the small column stub dimensions, and the distributed loading pattern creates conditions where two-way shear (punching) interaction governs over pure flexure. This is consistent with the known vulnerability of RC flat slabs to punching failure at slab-column connections^61^, and the observed crack patterns — diagonal cracks inclined at approximately 45° — are characteristic of this combined flexure-shear mechanism. Initial cracking generally emerged within the constant shear region specifically along the shear span—where the slabs developed inclined diagonal shear cracks. With the progressive increase of the applied load, both the number and width of cracks intensified, particularly on the tension face of the slabs, and were often accompanied by the formation of secondary vertical flexural-shear cracks. as loading advanced toward peak capacity, localized spalling of the concrete became evident, with the damaged areas extending gradually due to the degradation of the concrete cover and propagation of major shear cracks. the progressive evolution of these cracking patterns provides valuable insight into the underlying failure mechanisms of reinforced concrete slabs and highlights the varying effectiveness of the applied strengthening techniques in controlling crack initiation, propagation, and the extent of ultimate damage.

### Practical design recommendations

Based on the experimental and parametric findings, the following design guidance is proposed for engineers considering blast furnace steel slag concrete as a strengthening overlay for RC flat slabs: (1) Optimal overlay thickness: A slag concrete overlay of 20–30% of the original slab thickness (approximately 20–30 mm for a 100 mm slab) is recommended to maximize load capacity improvement while avoiding the adverse self-weight effects observed at 40–50% thickness. This 20–30% range is derived primarily from the numerical parametric study, since only one overlay thickness (20 mm, i.e. 20% of the 100 mm slab) was tested experimentally; it should therefore be treated as an indicative range requiring experimental confirmation at additional thickness levels, consistent with future-work. (2) Material specification: Compressive strength of slag concrete overlay should target ≥ 36 N/mm² based on the mix proportions in Table 8. (3) Aspect ratio considerations: For rectangular slabs, aspect ratios exceeding 1.4 result in significant reductions in ultimate load (≥ 55% loss compared to square slabs); designers should account for this through appropriate reinforcement or overlay adjustment. This aspect-ratio guidance is likewise derived from the numerical parametric study, since only one aspect ratio (1500 × 1500 mm) was tested experimentally, and should therefore be treated as an indicative limit pending experimental validation across a range of rectangular geometries. (4) Corrosion limits: Slabs with estimated bottom mesh corrosion exceeding 25% of diameter loss require immediate intervention, as load capacity falls below 53% of an intact slab. This corrosion threshold is likewise a numerically-derived indicative limit, based on the parametric FE corrosion model described previously, and should be corroborated by future experimental corrosion testing. (5) Surface preparation: Application of a bonding agent (e.g., epoxy-based) is recommended before casting the overlay to ensure composite action.

## Conclusion

This study presented a comprehensive experimental and numerical investigation into the strengthening of reinforced concrete flat slabs using blast furnace slag concrete as a sustainable alternative to conventional strengthening techniques. Five full-scale slab specimens (1500 × 1500 × 100 mm) were tested under static loading conditions, including one control slab and four slabs strengthened using different methods: FRP sheets, steel fiber-reinforced concrete, high-performance concrete, and slag concrete overlay. The experimental program was complemented by finite element modeling using ANSYS software to validate the experimental results and conduct parametric studies examining the effects of strengthening layer thickness, slab rectangularity ratio, and bottom reinforcement corrosion on structural performance. Based on the experimental testing, numerical simulations, and observed failure modes conducted in this study, the following key conclusions can be drawn:

- Strengthening slabs using slag concrete results in a significant increase in ultimate load capacity by approximately 29.9% and an improvement in the first crack load by 15.7% compared to the control slab.
- The use of slag concrete led to a reduction in ultimate deflection by 56.4% compared to the control slab.
- Compared to slabs strengthened using a 4 mm FRP layer, the application of slag concrete results in a decrease of approximately 12% in ultimate load capacity and 10% in ultimate deflection, while offering significant sustainability advantages.
- The developed FE model showed good accuracy with experimental results, with an average of 94% for ultimate load and 91% for ultimate deflection across all five tested specimens, confirming the reliability of the model for parametric investigation.
- Increasing the rectangularity ratio from 1.2 to 2.0 causes a decrease in ultimate load capacity by 73% at the same deflection level.
- Corrosion of the bottom steel mesh from 25% to 100% causes a reduction in ultimate load from 233 kN to 61.5 kN, corresponding to a reduction that increases from 0% to 73.6% compared to the intact slab without steel mesh loss.
- Strengthening slabs using slag concrete is superior to strengthening using ordinary concrete by up to 35% in ultimate load capacity.
- Increasing the strengthening layer thickness beyond 30% of the slab thickness reduces ultimate load capacity, first crack load, ultimate deflection, toughness, and post-cracking stiffness due to the adverse effect of increased self-weight.
- All tested slabs exhibited a combined flexural and shear cracking failure mode. Initial diagonal shear cracks developed along the shear span, followed by progressive widening and spalling toward ultimate failure. The strengthened slabs (S3–S5) demonstrated improved crack control and narrower crack widths at equivalent load levels compared to the control slab, confirming the effectiveness of the slag concrete overlay in delaying crack propagation and enhancing overall structural safety.

## Limitations and future studies

While this research provides valuable insights into the use of slag concrete for strengthening RC slabs, several limitations should be acknowledged, which in turn suggest important directions for future investigation. The experimental program was limited to five full-scale slab specimens with fixed dimensions (1500 × 1500 × 100 mm) tested under static loading conditions only, whereas real-world structures experience cyclic, impact, and fatigue loading under various boundary conditions beyond the simply supported configuration examined herein. Only one specimen was tested per strengthening method, with no replicate or companion specimens and no material-batch variability testing conducted, owing to the scale and cost constraints typical of full-scale RC slab testing; as a result, experimental variability could not be directly quantified, and the comparative percentage differences reported among S2–S5 should be regarded as exploratory/indicative rather than statistically conclusive. Long-term durability aspects, including environmental exposure effects (freeze-thaw cycles, chemical attack, carbonation), moisture ingress, and sustained loading over extended periods, were not investigated, nor was direct bond strength testing between the existing concrete substrate and the slag concrete overlay conducted. In addition, the finite element validation performed in this study was based on ultimate load and deflection together with a qualitative comparison of crack patterns; strain gauges or other direct stress-measurement instrumentation were not included in the experimental program, so quantitative validation of internal stress distributions could not be performed. The finite element model further assumes a perfect bond between reinforcement and concrete and represents reinforcement corrosion as a uniform reduction in bar diameter, and therefore does not capture bond-slip behavior, bond degradation, non-uniform pitting corrosion, or corrosion-induced cover cracking. Future research should therefore focus on: (1) investigating the performance of slag-strengthened slabs under dynamic and cyclic loading conditions, including seismic excitation and fatigue scenarios; (2) conducting accelerated aging tests and long-term exposure studies to evaluate durability under various environmental conditions; (3) performing comprehensive bond strength characterization using direct shear tests, pull-off tests, and interfacial fracture mechanics approaches; (4) expanding parametric investigations to include different reinforcement ratios, concrete grades, support conditions, and combined deterioration mechanisms; (5) implementing field applications with long-term structural health monitoring to validate laboratory findings; (6) optimizing partial slag replacement levels to balance mechanical performance, workability, cost, and environmental impact; (7) conducting life-cycle cost and environmental impact analyses comparing slag concrete to conventional strengthening methods, including an assessment of potential environmental leaching from the slag aggregate; (8) developing practical design guidelines compatible with existing building codes; (9) exploring hybrid strengthening systems combining slag concrete with FRP reinforcement or steel fibers; and (10) investigating punching shear behavior, as flat slabs are particularly vulnerable to this failure mode at slab-column connections; (11) testing at least three overlay thickness levels experimentally to directly validate the numerical thickness sensitivity results reported in this study. These research directions will contribute to establishing slag concrete as a validated, sustainable, and economically viable strengthening solution for aging reinforced concrete infrastructure while promoting circular economy principles in the construction industry; and (12) instrumenting future specimens with strain gauges or digital image correlation to enable quantitative validation of finite element stress distributions, and incorporating bond-slip and degraded-bond corrosion interface elements into future finite element models.

## Data Availability

All data generated or analyzed during this study are included in this published article.
